# Digitizing the Trails Making Test for Automatic Detection of Cognitive Impairment: Validity and Reliability

**DOI:** 10.1002/alz.089997

**Published:** 2025-01-03

**Authors:** Claudio Toro‐Serey, Connor Higgins, Russell Banks, Ali Jannati, Sean Tobyne, John Showalter, Alvaro Pascual‐Leone

**Affiliations:** ^1^ Linus Health, Boston, MA USA; ^2^ Michigan State University, East Landing, MI USA; ^3^ Harvard Medical School, Boston, MA USA; ^4^ Hebrew SeniorLife, Boston, MA USA

## Abstract

**Background:**

The Trail Making Test (TMT) is one of the tests most commonly included in neuropsychological batteries to assess executive functions. A digitized version of TMT would provide objective measurements of time to complete (TTC) and total number of errors (TE) with minimal training, and eventually enable process metrics including detailed evaluation of drawing behavior. Here we evaluated the test‐retest reliability and validity of a new digital TMT Part B (dTMT‐B).

**Method:**

We examined cognitively unimpaired participants from two clinical trials: DCT216 (N = 182, age mean±SD = 64.8±7.5; 60% female; years of education mean±SD = 14.4±2.5; 20% Hispanic; 3‐5 weeks between tests) and Bio‐Hermes‐001 (N = 342, age mean±SD = 70.5±6.4; 63% female; years of education mean[IQR] = 16[14‐23]; 6% Hispanic). For both TTC and TE, reliability was assessed via intraclass correlation coefficients (ICC 3, k) in DCT216. Internal consistency was evaluated using Cronbach’s alpha. We assessed validity by combining visit 1 of DCT216 and Bio‐Hermes‐001 and performing a multiple linear regression predicting TTC using predictors for study, age, sex, education, ethnicity, and interactions between study and each demographic. Specifically, we calculated the coefficients for age (key demographic in paper‐based TMT) and for demographic performance across both studies.

**Result:**

ICCs showed great reliability for TTC (0.87, 95% CI = 0.83 ‐ 0.90, p < 0.0001) and good reliability for TE (0.65, 95% CI = 0.53 ‐ 0.74, p < 0.0001). Cronbach’s alpha showed good internal consistency across these metrics as well (0.68). For validity, we found the expected significant main effect of age (T = 7.48, *p* < 0.001) and no significant interactions (*p* > 0.05), suggesting similar trends with paper‐based TMT‐B and consistent TTC values across studies.

**Conclusion:**

dTMT‐B is a valid and reliable digital implementation of the commonly used, paper‐based TMT Part B.

